# Dietary Risk Factors for Sporadic Creutzfeldt-Jakob Disease: A Confirmatory Case-Control Study

**DOI:** 10.9734/BJMMR/2014/7209

**Published:** 2014-02-01

**Authors:** Zoreh Davanipour, Eugene Sobel, Argyrios Ziogas, Carey Smoak, Thomas Bohr, Keith Doram, Boleslaw Liwnicz

**Affiliations:** 1 Department of Neurology, Loma Linda University School of Medicine, Loma Linda, CA, USA; 2 Department of Neurology, Keck School of Medicine, University of Southern California, Los Angeles, CA, USA; 3 Department of Preventive Medicine, Keck School of Medicine, University of Southern California, Los Angeles, CA, USA; 4 Department of Internal Medicine, Loma Linda University School of Medicine, Loma Linda, CA, USA; 5 Department of Pathology, Loma Linda University School of Medicine, Loma Linda, CA, USA

**Keywords:** Sporadic Creutzfeldt-Jakob disease, dietary risk factors, confirmatory case-control study, prion diseases, neuroepidemiology

## Abstract

**Aims:**

This study’s primary purpose was to determine whether earlier findings suggesting an association between sporadic Creutzfeldt-Jakob disease (sCJD), a transmissible spongiform encephalopathy of humans and specific dietary components could be replicated. The *a priori* hypotheses were that consumption of (i) foods likely to contain organ tissue and (ii) raw/rare meat are associated with increased sCJD risk.

**Study Design:**

Population-based case-control study.

**Place and Duration of Study:**

Department of Neurology, School of Medicine, Loma Linda University, Loma Linda, CA, USA; 4 years.

**Methodology:**

An 11-state case-control study of pathologically confirmed, definite sCJD cases, matched controls, and a sample of control-surrogates was conducted. Ninety-six percent (106/110) of the case data was obtained in 1991-1993, prior to variant CJD publicity.

**Results:**

Using control self-responses, consumption of hot dogs, sausage, pepperoni, kielbasa, “other” canned meat, poultry liver, any stomach/intestine, beef stomach/intestine, any organ tissue, and beef organ tissue was individually associated with increased sCJD risk; odds ratios (OR) ranged from 2.4 to 7.2 (0.003 <*p*<0.025). Rare/raw meat consumption was associated with sCJD (OR=2.0; *p*<0.05). Greater consumption of hot dogs, bologna, salami, sausage, pepperoni and kielbasa was associated with significantly higher risk. The OR for gizzard consumption was 7.6, *p*<0.04. Bologna, salami, any liver, beef liver and pork stomach/intestine were marginally associated with sCJD: ORs ranged from 1.7 to 3.7; 0.05 <*p*< 0.10. Brain consumption was not associated with an elevated risk. Analyses using control-surrogate data indicate that use of the control self-responses did not bias the results away from the null hypothesis.

**Conclusions:**

The *a priori* hypotheses were supported. Consumption of various meat products may be one method of transmission of the infectious agent for sCJD.

## 1. INTRODUCTION

Sporadic Creutzfeldt-Jakob disease (sCJD) is a fatal transmissible spongiform encephalopathy (TSE, prion disease) of humans. In 1979, Hadlow et al. [1] reported that the infectious agent (prion proteins or PrP^Sc^; Sc stands for scrapie, a prion disease in sheep and goats) was found in non-neural tissue, e.g., stomach, intestine, and the lymphatic system, even prior to clinical symptoms. More recently, Bosque et al. [2] found PrP^Sc^ in the skeletal muscle of mice inoculated with murine PrP^Sc^. Furthermore, they showed that substantial titers can be propagated within the skeletal muscle. Bosque et al. called for a concerted effort to determine the distribution and concentration of prions in animals (e.g., cows, sheep, deer) in the human food chain. Andréoletti et al. [3] investigated the prion distribution and titers in sheep which were either naturally or experimentally infected with scrapie. They found accumulation of PrP^Sc^ in muscles, with the titer being up to 5,000 times lower than in brain. However, as noted by Andréoletti et al. [3], van Keulen et al. [4] found much higher titers of PrP^Sc^ in lymph nodes located within muscles of scrapie-infected sheep. We note that the quantity of animal muscle consumed by humans is probably well over 5,000 times higher than the quantity of brain consumed.

In 1985, we reported on dietary risk factors associated with sCJD in a case-control study of 26 sCJD patients and 40 hospital and family controls from 5 states in the USA [5]. Combining the dietary findings with the findings of our report [6] of weak links in the human food chain (e.g., sheep raisers’ lack of awareness of scrapie in epidemic areas, the preclinical presence of the infectious agent(s) in non-neural organs of infected animals and the use of organs in certain meat products), we suggested that sCJD might be acquired through ingestion of contaminated food products, particularly processed foods which are likely to contain organ tissue. Four other epidemiologic studies of sCJD with dietary information were published prior to 1990 [7-10]. Two other studies were published in 1998 [11] and 2009 [12]. The findings of these studies are considered in the Discussion section.

Transmissible spongiform encephalopathy (TSE) has been found naturally in animals other than sheep, including goat, mule deer, elk, and mink. Experimentally, TSEs have a wide range of hosts [13-17], including pigs [18-20]. Research suggests that fish may be capable of harboring the infectious agent [21]. It should be noted that farm-raised fish, e.g., salmon, are fed meat byproducts. TSE has been reported in chickens [22-24].

The epidemic of bovine spongiform encephalopathy (BSE) in England, thought to have been initiated by scrapie-contaminated feed [25], the feeding of bovine products to cows, and a change in the rendering process in the late 1970s [26], convincingly demonstrates probable transmission of TSE across the species barrier by contaminated food products. BSE-contaminated feed has caused TSEs in several different species of zoo animals, including primates, and in domestic cats. Two lemurs (a small primate), one fed 0.5 g of BSE-infected cow brain once and one fed twice were found to be infected 5 months later [27]. (However, marmosets, another small primate, which may have been fed BSE-contaminated feed for 5-10 years have not developed histologically identified spongiform encephalopathy, although sCJD has been successfully transmitted to them [28]). There have been five reported outbreaks of TSE at mink farms in the US, each apparently due to contaminated feed, possibly from downer cows [29-31]. Mink have developed spongiform encephalopathy from oral inoculation with BSE-contaminated feed [32]. However, oral transmission of TSE from mink to cows has not been demonstrated [33]. Recently, it has been shown that at least some mammalian scavengers are apparently not susceptible to chronic wasting disease (CWD), a TSE of white-tailed deer and other cervids, after scavenging white-tailed deer carcasses in an area of Wisconsin where CWD is endemic [34]. There is convincing evidence that variant CJD (vCJD) in England and other countries and BSE are caused by the same agent, thereby establishing that transmission to humans has occurred [35-38]. Given the wide geographic distribution of the vCJD cases within Great Britain, the lack of contact with infected cows of most cases, and oral transmission to primates, transmission to humans has probably occurred through BSE-contaminated food [35-38].

### 1.1 Possibly Porous Species Barrier

The hypothesized species barrier may be more porous than previously thought. The question of dietary transmission of sCJD to humans is contentious [e.g., 11,26]. However, laboratory evidence provides credence to the possibility of such transmission, albeit somewhat inefficiently, e.g., (1) an *in vitro* study has identified a specific biological process by which human PrP^Sc^ may be chaperoned *in vivo* across the human intestinal epithelial cell barrier [39]; (2) it is now well-established that animals which do not manifest clinical signs of disease or only temporarily manifest such signs can harbor sufficiently high titers of infectious prions for disease to be transmitted at least through inoculation [40,41]; (3) prions can gain infectivity ability through passage from one animal to another [42] and (4) a second strain of prions in cattle has been identified which has molecular similarities with a specific subtype of sCJD [43]. It is also important to note that different prion strains, while derived from the same species-specific prion proteins, have different conformations which play an important role in the degree of infectivity, varying clinical signs and symptoms, and progression. Current evidence indicates that there are several different human PrP^Sc^ conformations [44]. These recent findings are discussed immediately below.

#### Crossing the Intestinal Epithelial Cell Barrier

Using an *in vitro* model of the human intestinal epithelial cell barrier, Mishra et al. [39] found that PrP^Sc^ is chaperoned by ferritin across the barrier. They concluded that, due to ferritin’s considerable homology across species, their study indicates that “PrP^Sc^-associated proteins, in particular ferritin, may facilitate PrP^Sc^ uptake in the intestine from distant species, leading to a carrier state in humans” or animals in the human food chain.

#### Subclinical Disease with Infectivity

Thackray et al. [40] studied two mouse-adapted scrapie strains (ME7 and RML) which were intracerebrally injected in a specific transgenic mouse model (Tga20) and two species of wild-type mice. Low doses of the inoculum caused the oscillation of appearance and then disappearance of clinical signs, sometimes for many months but did not induce terminal disease. The authors refer to this status as subclinical disease. PrP^Sc^ was identified in the brains of these mice at similar concentrations as found in mice with terminal disease. All titers were higher than contained in the original inoculate. Brain homogenates from these mice contained as much or nearly as much infectivity as brain homogenates from mice with terminal disease. This was determined by passage in Tga20 mice. When inoculated with brain homogenates from non-infected wild-type mice, none of the Tga20 mice developed disease. Thus, the brains of mice with subclinical disease contained sufficient quantities of PrP^Sc^ to induce terminal disease.

Hill et al. [41] studied a different strain of hamster prions considered nonpathologic to mice. When inoculated, the mice developed high titers of PrP^Sc^ in their brains, but were without clinical symptoms and had a normal lifespan. Upon transmission to a second generation of mice or to hamsters, all animals eventually developed clinical prion disease. Hill et al. suggested that there might be evolution of the PrP^Sc^ strain and that their data “seriously question our current understanding of species barriers”. We note that other researchers have also identified subclinical disease along with infectivity [45-47].

#### Gain in Infectivity with Transmission

Work by Race et al. [42] also suggests that apparently dormant PrP^Sc^ can evolve into a virulent form. They studied mice inoculated with a specific strain of hamster PrP^Sc^. They found that, despite not developing clinical disease, the brains and spleens of these mice remained infectious for the lifetimes of the mice. Furthermore, there was no evidence that the hamster PrP^Sc^ replicated within the brains for a period of at least one year. After this period of persistence, replication occurred and new strains were identified which caused disease in mice. Thus, new strains of PrP^Sc^ might develop in transmission between sheep (scrapie), deer or elk (chronic wasting disease) and cows (BSE). Some of these strains might be more easily transmitted and virulent to susceptible humans through diet.

#### Second TSE Strain in Cattle and sCJD

Casalone et al. [43] have recently identified a second TSE of cattle, with a molecular signature similar to a particular strain of sCJD (M/V2 CJD): type-2 PrP^Sc^ with a methionine/valine (M/V) polymorphism at codon 129 of the human prion protein gene. To date, this strain has been identified in two cattle. Casalone et al. have named the disease associated with this new prion strain bovine amyloidotic spongiform encephalopathy (BASE). The authors point out that there are differences in the distribution of PrP^Sc^ in BASE and M/V2 CJD. In M/V2 CJD, the largest amount of PrP^Sc^ is found in the cerebellum, brainstem and striatum, while in BASE the predominant areas are the thalamus and olfactory regions. Casalone et al. suggest that lack of substantial involvement of the motor dorsal nucleus of the vagus (MDNV) and the brainstem in BASE indicates that the route of transmission of BASE is perhaps not the alimentary tract. On the other hand, the brainstem is involved in sCJD. Unfortunately, no information concerning MDNV involvement in human sCJD appears to be available. We note that, given Casalone et al.’s suggestion [43], involvement of the brainstem would indicate that transmission could be through the alimentary tract.

#### Colitis and Increased Susceptibility of Mice to Oral Prion Infection

A recent study by Sigurdson et al. [48] has convincingly shown that C57BL/6 mice, after developing moderate colitis due to exposure of an attenuated strain of Salmonella, had more than double the usual susceptibility to oral prion infection and experienced a somewhat accelerated disease development. The authors conclude that “moderate intestinal inflammation at the time of prion may constitute one of the elusive risk factors underlying the development of TSE”.

In a review article, Collins et al. [49] concluded convincing evidence that high titers of infectious PrP^Sc^ can be present in asymptomatic animals. Their conclusion was that these findings “challenge previous ideas of species barrier”.

## 2. MATERIALS AND METHODS

This study was approved by the Institutional Review Board (IRB) of the Loma Linda University School of Medicine. The study participant subjects have signed the IRB approved informed consent form.

### 2.1 Study Design, Subjects and Controls

In this case-control study, cases and controls were ascertained from 11 states in the USA which were not included in our earlier case-control study [5]. These 11 states are Alabama, California, Florida, Georgia, Illinois, Louisiana, Michigan, Minnesota, Mississippi, Tennessee, and Texas. They were chosen because of their size; combined, they contain about 40% of the US population. Only neuropathologically confirmed CJD patients diagnosed between 1979 and 1990 were eligible for the study. Potential CJD cases were identified through systematic inquiries of hospitals and neuropathologists and from death certificates. The process of identifying subjects with a neuropathologic confirmation of CJD (definite sCJD) has been detailed elsewhere [50,51]. The criteria for definite sCJD were as follows: neuropathologically confirmed spongiform encephalopathy and progressive dementia with at least one of the following features: myoclonus, pyramidal signs, characteristic electroencephalogram, cerebellar signs, or extrapyramidal signs. Nearly 1000 possible cases were identified, of whom 189 had neuropathology reports and/or slides and tissue blocks which were available. The study neuropathologist (BL), neurologist (TB) and internist-geriatrician (KD) examined these materials and confirmed 162 to be definite CJD cases. With these data, we estimated that the incidence rate for the years 1986-88 in the study area was 0.83 per million populations, age-adjusted to the 1990 census [51]. No nosocomial CJD cases were identified. Familial CJD cases, defined as a case who had a blood relative with at least suspected CJD, were excluded. Thus, only sporadic CJD (sCJD) cases were used in the analyses.

Up to two controls from the general population for each case were obtained by random-digit-dialing. Matching criteria were date of birth (10 years earlier to 2 years after the case’s birth), gender, ethnicity (African American, White, Hispanic, Asian) and geographic area of residence 2 years prior to the onset of disease. Controls were, on average, 3 years older than their matched cases. A non-symmetric age matching interval was used because precise age-matching was not financially feasible and it was important that the controls had, at least on average, as much opportunity of exposure as the matched case. Allowing the controls to be, on average, somewhat older than the cases is conservative because they then have had more opportunity for exposure and the resulting odds ratio estimator may thus be somewhat biased downwards towards the null hypothesis of no increased risk.

### 2.2 Random Digit Dialing

The study began in 1990 and interviews began in 1991. After data were collected about a case, efforts began to recruit and interview at least one matched control, using random digit dialing. The area code and the first four digits of the telephone number of the case two years prior to disease onset were used to obtain controls. Random 3 digit numbers were generated for each case. The numbers were called in the order in which they were generated. However, before going on to the next number, 9 attempts had to be made: 3 times per day (morning, afternoon, evening) for 3 days, one of which was on a weekend. Nine rings constituted an attempt. It took about 30 hours of calling to find a matched control.

### 2.3 Interviews

Due to the nature of the disease (i.e., progressive dementia and death usually within one year), the most knowledgeable surrogate was interviewed for each case. Controls provided self-response data. For a subsample of controls (n=34), a surrogate was also interviewed. Thirty-two (32) of these surrogates provided sufficient dietary information for analysis. Interviews were conducted by telephone, which provided an efficient and feasible data collection method for a study which covered large geographical areas in different states in the US. Interviewees were sent materials describing the categories of information of interest so that they could prepare for the interview. Signed informed consent forms were obtained.

### 2.4 “Blinded” Interviewers

The interviewers were blinded as to the study subject matter, disease of interest, and case-control status. They did not associate with the other study staff. They knew only that the study was health-related and that sometimes the interview concerned the person being interviewed and sometimes it concerned another person. All interactions with the sCJD case families and the controls leading up to the interviews were conducted by the project coordinator, not an interviewer. The project coordinator requested that the informant not discuss the illness or the study with the interviewer. Frequent meetings and discussions with the interviewers confirmed their blinded status.

### 2.5 Dietary Exposures

The questionnaire was similar to the one used in our original CJD study [5]. It was, however, more focused. A series of questions asked whether specific food items were ever consumed up to six months prior to sCJD onset for cases. The same cutoff year was used for the corresponding matched control(s).

The interviewers asked about the frequency of “usual” consumption of each food item. Specifically, the interviewer asked “How often did (name) usually eat each of the following foods up to (year) when (he/she) was (number) years old?” For controls, “name” and “he/she” were replaced by “you”. Frequency was divided into 7 categories, from 5-7 times per week down to less than 1 time per year, and “never”. There was also a “do not know” category. In trend analyses, if the frequency of consumption was 2 times per month or more, consumption was classified as ‘high’, consumption less than 2 times per month was classified as ‘low’. These divisions were determined *a priori* and not as a consequence of preliminary data analyses.

### 2.6 Statistical Analyses

Control self-responses and control–surrogate responses for ‘ever’ versus ‘never’ consumption of specific food items were used to investigate whether surrogate responses tended to provide underestimates or overestimates of consumption of food items. The concordance rates for consumption of specific food items were determined, and the relative frequencies of positive responses for control self-responses and control–surrogate responses were compared. If surrogates tended to provide underestimates of consumption, the use of case–surrogate responses and control self-responses would likely result in a bias towards the null hypothesis. Alternatively, if surrogates tended to provide overestimates, the bias would be upwards, away from the null hypothesis. Case-control matching was retained in all ‘ever’ versus ‘never’ analyses using control self-responses and control–surrogate responses when the analysis was limited to cases with controls. In addition, analyses were repeated without consideration of the matching so that the data from the 25 cases without a control could be used. Odds ratio analyses using control–surrogate responses and analyses without matching were also used to investigate possible odds ratio estimator bias. ‘No consumption’ was the base from which odds ratios were calculated.

No multivariate analyses are presented because the odds ratios of the individual food items were of specific interest. The primary food items were chosen *a priori* because they were organs or were likely to contain organ parts. Other animal food items, e.g., bacon, were chosen because of the results of our earlier study. It is important to analyze each of these food items individually. Because this was a confirmatory study with *a priori* specified hypotheses, it is appropriate to use one-sided statistical tests (p-values) for the odds ratio estimates associated with these hypotheses. However, two-sided 95% confidence intervals for the odds ratios are used because they are standardly provided. Therefore, at times the (one-sided) statistical test for an odds ratio has a p-value below 0.05, but the confidence interval contains 1.0. It should be noted that a one-sided p-value below 0.025 translates into a two-sided p-value below 0.05.

Exact conditional logistic regression was used for estimating the odds ratios for the ‘ever’ versus ‘never’ (dichotomous) comparisons and 95% confidence intervals (CIs) [52,53]. One degree of freedom chi-square trend tests (2-sided) for ‘high’ versus ‘never’ and ‘low’ versus ‘never’ consumption of specific food items were used. The same statistical tests were used for the other dietary items (not *a priori* specified), but were 2-sided. All data analyses were conducted using SAS (SAS Institute, Inc., Cary, NC).

## 3. RESULTS

### 3.1 Description of Cases and Controls

One hundred sixty-two neuropathologically confirmed cases were identified. Nineteen (12%) families could not be located and 23 (14%) families declined to participate. Thus, interviews from surrogates of 120 (74%) of the 162 neuropathologically confirmed Creutzfeldt-Jakob disease (CJD) cases were obtained. One hundred ten (92%) of the 120 CJD cases were sporadic. Among the sporadic cases, 45 (40.9%) were women. The mean age-at-onset of symptoms was 63.6 years, with a standard deviation of 8.8 years. The age-at-onset distribution was rather symmetric, with a range from 34 to 80.3 years. There was essentially no difference in the onset distribution between men and women. The mean duration of disease (clinical onset to death) was 8.5 months. Women had a longer mean duration than men, 10.4 versus 7.1 months.

Twenty-nine cases had two matched controls and 56 cases had 1 matched control. Thus 85 (77%) of the 110 sporadic CJD cases had at least one control. The cases without a control were, on average, 4 years older at clinical onset and had a slightly narrower age-at-onset standard deviation than the cases with at least one control. These cases also had a shorter mean duration of disease: 7.3 versus 8.7 month mean durations. Two-thirds of the controls were older than their matched case. The controls were, on average, born 3.0 years earlier than their corresponding case. 93.7% of both the cases and controls were “White”. There were no differences in the distributions of country of origin of the parents of the cases and controls. Forty percent of the controls and 45.5% of the cases had no more than a high school education; 6% of the controls and 11.6% of the cases had some graduate education. Nearly two-thirds of the cases were urban/suburban residents two years prior to disease clinical onset. Because controls had the same area code and prefix as the cases, the same percentage of controls were urban/suburban residents. Table 1 displays some of this information.

### 3.2 Consumption of Specific Food Items and Rare/Raw Meat

Table 2 provides the frequency data for consumption of the specific food items about which information was requested and the level of cooking of meats. Information about a wide range of items was sought. The rates of missing information for cases and controls were relatively low (below 10%), except for raw milk (11%), smoked pork (12%), kishka (12%), scrapple (22%), sausessen (20%), rabbit (11%), and “other” canned meat (11%) among cases. Controls more frequently ate 29% of the food items in Table 2 than cases.

### 3.3 Analyses for Organ Tissue Consumption and Meat Preparation

Table 3 presents the odds ratio estimates, 1-sided p-values and 2-sided 95% confidence intervals for ‘ever’ versus ‘never’ consumption for the *a priori* identified food products likely to contain organ tissue, for specific organ tissue and for ‘rare/raw’ versus ‘medium/well done’ meat consumption. Among food items likely to contain organ tissue, consumption of hot dogs, sausage, pepperoni, kielbasa and “other” canned meat were all significantly associated with sCJD at below the p=0.025 level, with ORs between 2.4 and 7.2. Consumption of bologna and salami had ORs of 1.7 (p<0.07) and 1.9 (p < 0.055), while consumption of kishka, scrapple and meat spreads had ORs of 2.1 (p=0.17), 1.04 (p=0.56) and 1.3 (p=0.25), respectively. Evidently, no subject ate sausessen. The 95% CIs were narrow except for hot dogs and sausage, because of the very high proportion of both cases and controls who had consumed these food items.

Consumption of any internal organ tissue, based on consumption of a specific named organ, had an OR of 2.5 (p<0.004, 95% CI: 1.3-5.3). Consumption of beef internal organ tissue had an elevated OR: 3.2 (p<0.003, 95% CI: 1.4-8.4). Consumption of any stomach/intestine had a significantly increased OR of 3.7 (p=0.006, 95% CI: 1.3-13.1). The odds ratios for consumption of beef and pork stomach/intestine were 4.5 (p=0.01, 95% CI: 1.2-25.4) and 3.7 (p<0.10, 95% CI: 0.6-37.6), respectively.

Consumption of any liver was marginally significant: OR=1.9, p<0.08, 95% CI: 0.8-4.9). Consumption of beef liver was also marginally significant, and consumption of poultry liver was significantly elevated (OR=3.1, p<0.007, 95% CI: 1.2-9.0). Consumption of pork liver was not significantly elevated. Consumption of heart, kidney or brain was not elevated among cases. Consumption of gizzard (poultry) had a high odds ratio, which was only marginally significant. The gizzard consumption rate among controls was only 1%. Eating rare or raw meat had a significantly elevated odds ratio (OR=2.0, p<0.05, 95% CI: 0.9-4.7).

### 3.4 Trend Analyses

Table 4 provides the trend analyses (risk associated with increasing consumption) for the *a* priori chosen food items likely to contain organ tissue for which high frequency consumption was at least somewhat common. For completeness, the individual odds ratios for ‘low’ versus ‘never’ and ‘high’ versus ‘never’, the 2-sided 95% CIs and the 1-sided p-values are also provided. The 2-sided significance levels (p-values) for trend for sausage and pepperoni consumption were both below 0.02. For hot dogs, salami, kielbasa and bologna consumption the 2-sided p-values were each below 0.01.

### 3.5 Analyses of Additional Food Items: 2-Sided Tests

Dietary information was inquired for several specific meat items, e.g., lamb chop, roast pork, corned beef, smoked fish, raw fish. Odds ratios were significant (2-sided tests) for only the following three items: lamb/mutton, smoked pork, and lobster: 3.1, 2.0, 2.3, respectively. Dietary histories of vegetable consumption were also obtained, specifically, potato, onion, carrot, and “other” root vegetables. “Other” root vegetables mentioned with some frequency were beets, turnips, yams/sweet potatoes, rutabagas, radishes and parsnips. Odds ratios were significant only for beets and turnips; they were both below 1.0.

### 3.6 Concordance Rates and Odds Ratio Estimators Based on Control Self-Responses and Control Surrogate-Responses

Exposure was sufficiently frequent for most of the specific food items of *a priori* interest to compare control self-responses and control-surrogate responses among the n=32 pairs. The concordance rates were generally above 60% (9 of 15 comparisons, Table 5). Controls self-reported more frequent consumption of hot dogs, bologna, salami, sausage, kielbasa, kishka, scrapple, meat spreads, any brain, any heart, any liver and any stomach/intestine than did their surrogates (Table 6). The rates were the same for pepperoni and “other” canned meat. Only for kidney was the surrogate reported rate greater than the control self-reported rate.

Table 6 provides a comparison of the odds ratio estimates for the *a priori* chosen food items using control self-responses with matched and unmatched analyses and using control-surrogate responses in a matched analysis. The unmatched analyses use all 114 sCJD cases. The odds ratio estimates using matched and unmatched analyses with control self-responses differed by 0.2 or less for 10 (38%) of the 26 food/preparation items. The matched analysis resulted in an odds ratio higher by more than 0.2 for 8 (31%) items and lower by more than 0.2 in the remaining 8 (31%) items. In general, the unmatched analyses had lower significance levels (i.e., smaller p-values), mostly because of the increased number of cases. Only pork and poultry organ tissue consumption changed from non-significance in the matched analysis to having 1-sided p-values below 0.05 and 0.10 in the unmatched analysis: ORs of 1.6 versus 1.8 and 1.4 versus 1.9.

The matched analyses with control-surrogate responses had many fewer case-control pairings than the matched analyses with control self-responses: 32 versus 85. However, for only 5 (19%) of the food items were the odds ratios using the control-surrogate response data lower by more than 0.1. These food items were salami (1.9 to 1.6), sausage (4.4 to 3.6), any kidney (1.2 to 0.2), beef liver (1.7 to 1.1) and pork stomach/intestine (3.7 to 3.0). For most of the other food items the increase in the odds ratio was relatively large, e.g., bologna (1.7 to 5.4), beef organ tissue (3.2 to 6.0) and any brain (0.7 to 2.5).

## 4. DISCUSSION

Below, we discuss bias considerations in the present study and in studies by other groups. Caution must always be exercised in evaluating the results of any epidemiologic study, whether case-control, cross-sectional, or prospective. Conclusions can be affected by problems in nearly all components of an epidemiologic study, including the information that is collected, the determination of case and control status (or disease incidence for prospective studies), the training and effectiveness of interviewers and other data collectors. This is why replication by different groups is generally essential.

### 4.1 Bias Considerations

#### 4.1.1 General Considerations

Potential bias in this study was minimized by (i) identifying as many of the pathologically confirmed definite cases of sCJD in a well-defined large geographic area and time period as possible, (ii) selecting controls through random digit dialing, (iii) matching controls to individual cases by gender, age, ethnicity and location of residence, (iv) keeping the interviewers blinded as to case-control status, the disease under study, and the hypotheses being investigated, (v) providing written materials to the respondents concerning the information to be requested so that they could prepare for the interview, and (vi) embedding questions relating to the *a priori* hypotheses among other questions. It was necessary to interview a surrogate for each case due to the nature of the disease, i.e., all cases had become demented and most had already died. (sCJD cases are generally demented before a clinical diagnosis can be made. However, a clinical diagnosis is not considered very reliable. Thus, surrogates would have been required even if cases were available for interview at the time of a clinical diagnosis).

Both differential and non-differential recall biases are potential problems in case-control studies. To help guard against these recall biases, case–surrogate and controls were provided information *prior* to the interview concerning the items to be queried, as mentioned above. This enabled them to think about the past and to ask other individuals if they were not fully knowledgeable about a subject. On the other hand, control-surrogates were not allowed to ask for information. Thus, case-surrogate information may be nearly as accurate as control information and more so than control-surrogate information. The questionnaire included items about occupations, hobbies, travel, residential locations, and medical history, and thus did not focus on diet. Information on approximate usual frequency of consumption of numerous food items was obtained.

##### Case-Surrogates Lacked Knowledge of Hypothesized Dietary Risk Factors

Until the late 1990s/early 2000s, physicians knowledgeable in the clinical diagnosis of CJD, researchers, and government officials by and large had discounted the idea that any form of CJD may be transmitted to humans through dietary exposure, even BSE-contaminated foods. vCJD was first identified in 1996. Subsequent research has demonstrated that BSE and vCJD are caused by the same infectious agent and an oral route of transmission is now accepted.

However, almost all case data (96%) from the present study were collected in 1991-1993; data for 4 cases were collected in early 1994. No case-surrogate mentioned or inquired about dietary risk factors in conversations with the PI or project coordinator. Many case-surrogates asked about the causes of CJD and were told that causes were unknown and that was why the study was being conducted. Thus, until the BSE epidemic received publicity after 1993, the families of CJD patients were unlikely to have had knowledge about any possible mode of transmission, including oral.

Consequently, it is unlikely that case-surrogate data were biased due knowledge of the dietary transmission hypotheses. This conclusion is bolstered by the fact that the odds ratio estimates using control self-responses for brain and raw milk consumption were below unity and only 3 *non-a priori* specified food items for which consumption data were obtained had a statistically significantly elevated risk: lamb/mutton (but not lamb chops or roast lamb); smoked pork (but not pork chops, roast pork, ham or bacon) and lobster. It should be noted that mutton comes from older sheep, which are at increased risk of having subclinical scrapie compared to younger sheep or lamb and smoked pork is not cooked at a high temperature.

#### 4.1.2 Recall Bias

As discussed above, we designed the data collection protocol to minimize recall bias and we were fortunate that 96% of the data collection for cases was prior to BSE publicity in the US and 100% was collected in 1991-1993 and early 1994. (vCJD was identified in England in 1996.)

Given that (i) a possible *a priori* dietary transmission hypothesis was unknown to our case–surrogate, (ii) the presence of organ parts in processed meats is generally not known by the public, and (iii) dietary questions were embedded in a detailed questionnaire covering numerous topics, recall bias is likely not to be a reason for our positive findings.

#### 4.1.3 Use of Control Self-Responses Is Likely Conservative

Analyses using control-surrogate responses usually resulted in larger odds ratios than analyses with control self-responses (Table 6). Thus it is likely that the control self-response data generally resulted in conservative odds ratio estimates because control self-responses should be more complete than control-surrogate responses for dietary items.

### 4.2 National CJD Surveillance Unit (UK) Case-Control Studies: Bias Considerations

#### Overview

The National CJD Surveillance Unit (UK), between 1990 and 1997 conducted case-control studies using definite and probable CJD cases, hospital-based controls, and ‘non-cases’, defined as subjects referred to the Unit as possibly having CJD but who, upon further review or examination, did not have CJD [54,55]. In the 4th (1995) and 6th (1997) annual reports, data were presented on the consumption of beef, venison (1995 only) and brain (1997 only) for ‘non-cases’ [54,55]. Only these three food items were selected because in 1995 stepwise backward multivariate odds ratio analyses for cases vs controls found beef and venison to be jointly significant, while similar analyses in 1997 found beef and brain to be jointly significant (S Cousens, personal communication, 1999). Odds ratios for cases vs ‘non-cases’ were reported for beef, venison and brain. The sample sizes were 150 cases and 51 ‘non-cases’ in 1995 and 206 cases and 80 ‘non-cases’ in 1997.

Based on a finding that these odds ratios were not significantly different from 1 (whereas the ORs for cases vs controls were significantly greater than 1), the authors of the reports concluded that the positive findings for cases vs controls may be solely due to recall bias. The 4th annual report findings (beef and venison) were referenced by van Duijn et al. [11] to substantiate their statement that “some of the positive findings in this study may largely or entirely reflect respondent bias”. The 6th annual report findings (beef and brain) were cited by Johnson and Gibbs as a reason for discounting diet as a risk factor [56].

#### Detailed Review

The CJD Surveillance Unit’s data collection protocol may have introduced serious bias in their data, which makes interpretation problematic. Perhaps the two most important problems are that (1) meat consumption, particularly beef, is a risk factor for many of the diseases of the ‘non-cases’ (see below) and (2) data collection was performed by research assistants and physicians who were neither blinded to case-control status nor to the opinion of the primary investigators that sCJD is unlikely to be transmitted through diet. Also, probable sCJD cases were included as cases, which due to misclassification, likely lowered the odds ratio estimates.

Important diagnostic information about the ‘non-cases’ is lacking in the CJD Surveillance Unit’s reports. However, perhaps up to 60% of the ‘non-cases’ were determined not to have CJD merely on clinical grounds and only a “significant proportion of these cases made a complete clinical recovery” [11]. Thus, there may actually be some CJD cases among the ‘non-cases’.

No judgment is made in the reports as to what proportion of the ‘non-cases’ had an illness or disease likely associated with fat or beef consumption. Information about the final clinical or pathologic diagnoses of the ‘non-cases’ is not provided but apparently the two most common primary diagnoses were Alzheimer’s disease (AD) and Lewy body disease, with other diagnoses such as cancer and infarct (S Cousens, personal communication, 1999). AD is difficult to distinguish from vascular dementia (VaD) in the absence of expert diagnoses, so it is likely VaD was also at least somewhat common among the ‘non-cases’. VaD and infarcts, either embolic or thrombotic, are clearly associated with increased cholesterol intake, e.g., beef consumption. In fact, dementia in general may be associated with meat consumption. The Rotterdam study of dementia found that high intake of total fat had a significantly elevated odds ratio of 2.4 (95% CI: 1.1-5.2) [57]. The ORs for saturated fat and cholesterol were elevated, but were marginally significant (0.05<p≤0.10). In a matched sub-study, the Adventist Health Study found that a history of “heavy” meat consumption had an OR for dementia of 2.99 (p<0.05) compared to vegetarians [58]. The CJD Surveillance Unit’s unmatched sub-study had limited power to detect an effect of “heavy” meat consumption. Thus, it is not surprising for the beef consumption of ‘non-cases’ to be elevated compared to controls.

From the reported data, the frequency of consumption of beef, brain and venison for cases and controls from 1990-1995 and 1995-1997 can be determined. For ‘non-cases’, only the frequency of consumption of beef for these time periods can be determined. In 1995, ‘non-cases’ were reported to have eaten beef more frequently than cases and thus the OR for ‘non-cases’ vs controls was higher than for cases vs controls. However, for venison, ‘non-case’ and control consumption were quite close (Table 7). In 1997, the rate of beef consumption for ‘non-cases’ was virtually identical to cases, while brain consumption for ‘non-cases’ was midway between cases and controls. The absolute and relative frequencies are provided in Table 7. These data indicate that, among both cases and ‘non-cases’, reported “high” beef consumption dropped 6.1% and 11.9%, while “low” consumption (baseline) increased 6.4% and 10.6%, respectively. “Moderate” consumption remained virtually unchanged. Among controls, reported beef consumption remained practically unchanged. The existence of vCJD was made public in 1996. If recall bias were a problem in the CJD Surveillance Unit’s study, one would have expected an increase in reported beef consumption, not a decrease, for the 1995-1997 period compared to the 1990-1995 period.

Within the case and control groups, reported rates of brain consumption were low and were essentially the same for the two periods. Reported venison consumption among cases changed very little between the two periods. However, among controls, there was a statistically significant (p=0.002) increase in the proportion who had ever eaten venison (Table 7). The break in the time periods was at the time that vCJD became known to the Surveillance Unit, which, given their ideas about dietary transmission, might have unconsciously influenced the depth of interviewing case–surrogates (less depth) and controls and control–surrogates (more depth) during the 1995-1997 period. Note that there was much more opportunity to probe for venison consumption, given the higher percentage of controls reported to have never eaten venison compared to cases.

### 4.3 Multiple Comparisons

In the present study, the processed food items which may contain bovine, sheep and porcine organ tissue and which showed an association with sCJD were chosen *a priori*, based on the results of our earlier study [5]. Analyses, using an index of the number of these *a priori* chosen food items consumed, showed increasing risk with increasing number of items consumed (data not shown). Given the consistency of the results, multiple comparisons therefore do not appear to represent a problem.

### 4.4 Codon 129 Homozygosity and Estimated Odds Ratios

Fortunately, oral inoculation appears to be an inefficient route of transmission of sCJD. Meat may generally be unlikely to contain high levels of the infectious agent. (However, meat products containing organ tissue are not necessarily well cooked and cases ate rare or raw meat more frequently than controls.) Homozygosity at codon 129 on the prion precursor protein gene is a susceptibility factor. About 85%-95% of the cases are homozygous at codon 129 [65-68]. Approximately 50% of the general population is homozygous at codon 129. It was not possible to determine the zygosity status of the cases in the present study.

The odds ratio estimates may be biased towards 1 because about 50% of the controls were likely heterozygous at codon 129, were consequently about 5.7 (85/15) to19 (95/5) times less likely to develop sCJD than homozygotes given the same exposures and thus their dietary history could have been comparable to the sCJD cases’ dietary histories without adverse effect.

### 4.5 Comparison with Previous Studies

Table 8 provides the odds ratio information for specific dietary items common to the earlier Davanipour et al. study, the present study, the van Duijn et al. study [11] and the Ruegger et al. study [12]. Results from the other earlier studies [7-10] are included if the data were collected and available. The results of the Davanipour et al. and van Duijn et al. studies are generally consistent. The Ruegger et al. study [12], despite likely using a questionnaire similar to that used by van Duijn et al. [11], was not congruent. Only the two Davanipour et al. studies included processed food items likely to contain organ tissue, except for sausage items in the van Duijn et al. and Ruegger et al. studies [11,12].

#### 4.5.1 Findings from Specific Studies

The *Bobowick et al. study* [7] simply asked about consumption of raw meat, raw seafood, and brain. No significant risk association with raw meat consumption was found. A significant association was found with consumption of raw seafood, but was discounted because it was limited to raw oysters in “almost all cases”. Consumption of brain in general was not statistically significant, but consumption of pig brain was marginally increased among cases (OR = 3.6, p = 0.07). We note that the Goldberg et al. study [8] found that among cases and controls who ate brain, the cases generally preferred light grilling and the controls generally preferred frying or simmering. Light grilling is more likely to leave the heat-resistant abnormal prion viable.

The *Kondo and Kuroiwa study* [9] reported on consumption of raw meat and brain. Only raw horse meat had been consumed, by 3 cases and 3 neighbor controls. No subject had eaten brains. The dietary data are discussed, but not presented in the *Harries-Jones et al. study* [10]. The authors state, however, that cases “tended” to eat less kidney and more tripe than controls and were “more likely” to have ever eaten other forms of organ tissue but there was “no clear evidence” of an increase in risk with an increase in consumption. The associations are referred to as “weak”. Statistical levels of significance were not reported.

The initial Davanipour et al. study [5] was relatively small. Twenty-six (26) CJD cases were ascertained in a five state area (Pennsylvania, New Jersey, Maryland, New York, and Delaware). (None of these states were involved in the 11 state study reported in this communication.) Twenty-three (23) were identified from the records of the Laboratory of Central Nervous System Studies, US National Institutes of Health. Three (3) additional CJD cases were identified through relatives of the original 23 cases. Twenty (20, 77%) had their diagnosis confirmed by pathologic findings and the disease was successfully transmitted to animals for 8 of these cases. Thus, 6 subjects were classified as “probable” CJD cases. Two cases were familial. Two types of controls were used: hospital controls; family controls. Hospital controls were matched by gender, age (±5 years), and date of case in-hospitalization diagnosis (as close as possible). Family controls were through case–surrogates (who supplied the case exposure information), with matching for age (± 10 years) and gender. Forty-five (45) food items were of interest. Categories were red meat, fowl, game, seafood, processed meat, organ meat, and dairy products. Major changes in diet were lacking according to the interviewees. ‘High’ and ‘low’ consumption were defined as consumption at least once a month and less than once a month, respectively. High, low and ever consumption were compared to no consumption. Analyses were conducted using family controls, hospital controls, and all controls. In addition to the results provided in Table 8, (i) high or ever consumption of deli/canned ham was statistically significant relative to family and hospital controls (p<0.05) and relative to the combined controls (p<0.01), roast pork (high or ever) was statistically significant (p<0.05) relative to hospital controls, (ii) hot dog consumption (ever vs. never) was significant relative to hospital controls (p<0.01) and marginally significant relative to the combined controls (p<0.10), (iii) pork chop consumption (high or ever) was marginally significant (p<0.10) for hospital controls and (high only) for the combined controls, (iv) smoked pork (high) consumption was marginally significant (p<0.10) relative to hospital controls only and (v) scrapple (high) was marginally significant (p<0.10) for the combined controls.

The raw data from the Kondo and Kuroiw [9], Harries-Jones et al. [10] and Davanipour et al. [5] studies have been combined for a meta-analysis by Wientjens et al. [59]. Neither the Bobowick et al. study [7], which did not use the Masters et al. diagnostic criteria [50], nor the Goldberg et al. study [8] were included. Dietary questions in the Kondo and Kuroiwa and the Harries-Jones et al. studies were not detailed and did not often overlap with the other two studies. The dietary data items in the meta-analysis were thus severely restricted. Consumption of ‘any organ meat’, although having different practical meanings in each study, was the only relatively common food item. Given the relative sizes of the studies in relation to the individual findings, consumption of ‘any organ meat’ was not elevated in the meta-analysis.

The *van Duijn et al. study* [11] was larger than the earlier studies, covered 6 European countries, and inquired about several dietary items: beef, veal, lamb, pork; sausage, tripe, kidney, liver, brain, eye; raw meat, raw fish; unspecified animal blood products; milk and cheese. Consumption of brain had a statistically elevated risk (OR = 1.7, 95% CI = 1.2-2.4). Consumption of raw meat also had an elevated risk (OR = 1.6, 95% CI = 1.2-2.2). Consumption of sausage, any beef, any veal, any lamb, or any pork was not associated with an increased risk. However, there was a statistically significant trend in risk with increased consumption of pork (p for trend 0.02). Consumption of tripe, kidney, liver or eye was not associated with an elevated risk. Consumption of raw fish had an odds ratio of 1.5, but was not significant. Data were missing for over 70% of the cases and controls for brain and raw fish consumption.

Data collection for the van Duijn et al. study [11] was between 1993 and 1995, mostly after the beginnings of the widespread concern about BSE. Perhaps somewhat over 50% of the data were collected prior to the identification of vCJD in 1995. The odds ratio estimate for brain consumption was higher (and statistically significant) than in any of the other studies (Table 8). [However, in the present study, when control-surrogate data are used, the OR for brain is 2.5 (Table 6), but not significant.] It is conceivable that case-surrogate responses in the van Duijn et al. study were influenced by the BSE concern. The authors state that when a matched analysis was performed, brain consumption was no longer significant (p=0.18). However, neither the OR nor the number of case-control pairs is provided. Assuming missing information was random among the case and control–surrogates, the expected number of case-control pairs with data is small, approximately 33.

#### 4.5.2 Consumption of Food Items Likely to Contain Organ Products

Only the earlier Davanipour et al. study [5] and the present study reported on consumption of processed food items, except for sausage in the van Duijn et al. [11] and Ruegger et al [12] studies. It was the results concerning processed food items in our earlier study which formed the basis for the *a priori* hypotheses for the present study. Organs themselves when directly consumed are likely to be cooked more thoroughly than when they form part of a processed food item. This is likely unknown to the consumer. For the processed food items, we found significantly increased risk associated with hot dogs, sausage, pepperoni, kielbasa and “other” canned meat. Bologna and salami consumption has a marginally increased risk, while, scrapple, meat spreads, and kishka had no increase in risk. Kishka had an odds ratio estimate of 2.1 but was rarely consumed by subjects in our study. In addition, we found significant trends in risk with increased consumption for the items with sufficient frequencies of high consumption for trend analyses. van Duijn et al. [11] reported that there was not a significant trend in risk associated with increased consumption of “meat products such as sausage or black pudding”. {Black pudding is a beef blood and lard product and thus does not belong to one of the food items constituting our *a priori* hypothesis. While there is concern about transmission of CJD through blood transfusions, there are no suggestive data [60-62].} Information as to what other meat products were included in the data collection was not provided. Only the data and odds ratio for ‘ever’ versus ‘never’ consumption of sausage were provided: OR = 1.2, with about 95.5% of cases and 94.5% of controls having ever consumed sausage. The ‘ever’ consumption rate is essentially identical for cases in the present study, but only 83% of the controls reported consumption; the OR was 4.4 (p<0.009). ORs for both low and high consumption (vs ‘never’ consumption) were statistically significant and there was a statistically significant trend (Table 4).

#### 4.5.3 Consumption of Pork

Using 3 levels of consumption, van Duijn et al. [11] found a statistically significant trend in risk (p<0.02) for consumption of any pork item. In the present study, using two levels of consumption, there was also a significant trend in risk (p<0.004; data not shown). Smoked pork consumption was also increased among cases in our present study. It has been demonstrated that pigs develop spongiform encephalopathy through inoculation of BSE-infected brain. Pigs are slaughtered while quite young. If a pig were to become infected, certain organ tissue could be contaminated, but clinical symptoms would be very unlikely.

#### 4.5.4 Consumption of Rare/Raw Meat and Raw Seafood

van Duijn et al. [11] reported odds ratios of 1.6 (95% CI: 1.2-2.2) for consumption of raw meat and 1.5 (95% CI: 0.7-3.2) for consumption of raw fish. In the present study, the odds ratios for consumption of rare/raw meat and raw fish were, respectively, 2.0 (95% CI: 0.9-4.7) and 2.4 (95% CI: 0.8-7.2). These estimates are not dissimilar from van Duijn et al.’s estimates. Currently, farm-raised salmon and perhaps other fish are fed meat byproducts, which may be contaminated [21]. Cuts of meat may contain brain and possibly other organ parts due to the use of pneumatic-actuated penetrating captive bolts to slaughter cattle and other large food animals [63].

## 5. CONCLUSIONS

In general, there is a contrast between the occurrence of CJD cases and the large number of individuals consuming these food items. It should be noted that TSE is a rare disease in animals. In addition to a low TSE exposure rate, TSE infectivity and viability are likely low. Moreover, human susceptibility to TSE may be related to rare genetic make-up. Consequently, it should not be surprising that sCJD is, fortunately, rare. Ecological and other issues are discussed in reference [69].

Over all, we found evidence that consumption of certain beef products may be associated with increased risk of sCJD and that consumption of certain items of pork and poultry were associated with an increased risk. In addition, many of the food items likely to contain brain, e.g., various sausages, may contain meat and organs from animals besides cows. This study should be interpreted as providing evidence that sCJD is, at times, a zoonotic disease, with possible transmission via dietary items. TSE occurs naturally in wide range of animal hosts, e.g., sheep, goats, bovine, mink, white-tailed deer.

It has been established that cows in Great Britain and perhaps elsewhere probably developed BSE by eating contaminated food products, e.g., sheep products. It has also been established that cows which consumed contaminated cow offal have developed BSE. Earlier, mink which ate contaminated feed, perhaps bovine, in the US developed a spongiform encephalopathy (TME). The literature has other examples of transmission of spongiform encephalopathy between species through contaminated feed. One reason for increased transmission of this disease by ingestion within and between species may be an unfortunate change in the processing procedures of offal during rendering, making it more likely that the infectiousness of the agent survives [26,70,71].

In summary, the findings in this study support the hypothesis that one way for humans to develop sCJD is consumption of animal products containing the infectious agent for TSE. Processed food items which may contain organ tissue products may represent a risk. Some of these food items are not sufficiently cooked.

## Figures and Tables

**Table 1:** Descriptive information for definite sporadic cases and controls

		**Controls**		
**Sample** **Size**	**Cases**	**Number of Matched Controls per Case**		
		**2**	**1**	**0**

	**110**	**29**	**56**	**25**

**Case Gender**		**Number of Subjects (Percent)**		

Female		45 (40.9%)		
Male		65 (59.1%)		

**Other Descriptive Information**				

Percent of Controls Older than Matched Case		67%		
Mean Age Difference: Controls – Cases		+ 3.0 Years		
Mean Age-at-Onset (SD) of Symptoms		63.6 (8.8) Years		
Range of Age-at-Onset of Symptoms		34.0 – 80.3 Years		
Mean Duration of Disease: Clinical Onset to Death				
All Cases		8.5 Months		
Female Cases		10.4 Months		
Male Cases		7.1 Months		
Clinical Onset & Disease Duration: Cases with no Control vs Cases with a Control				
Mean Age Difference		Cases with no Control – 4 Years Older		
Mean Duration of Disease		7.3 vs 8.7 Months		

**Education**		**Cases**		**Controls**

High School or Less		45.5%		40.0%
Some College Education		42.9%		54.0%
Some Graduate Education		11.6%		6.0%

**Case Residential Location 2 Years Prior to** **Clinical Onset**				

Urban/Suburban		67.7%		

Rural		32.3%		

**Table 2:** Descriptive statistics for dietary items*

Exposure Variable	Cases			Controls		

	Exposed	Not Exposed	Missing	Exposed	Not Exposed	Missing
Raw Milk	36 ( 42%)	40 (47%)	9 (11%)	60 (53%)	51 (45%)	3 (2%)

Lamb Chop	45 ( 53%)	37 (44%)	3 ( 4%)	70 ( 61%)	42 (37%)	2 (2%)
Roast Lamb	45 ( 53%)	37 (44%)	3 ( 4%)	63 ( 55%)	50 (44%)	1 (1%)
Lamb/Mutton	47 ( 55%)	35 (41%)	3 ( 4%)	39 ( 34%)	74 (65%)	1 (1%)
Any Pork	82 ( 96%)	2 ( 2%)	1 ( 1%)	105 ( 92%)	9 ( 8%)	0 (0%)
Pork Chops	80 ( 94%)	4 ( 5%)	1 ( 1%)	103 ( 90%)	11 (10%)	0 (0%)
Roast Pork	76 ( 89%)	8 ( 9%)	1 ( 1%)	94 ( 82%)	20 (18%)	0 (0%)
Ham	81 ( 95%)	3 ( 3%)	1 ( 1%)	102 ( 89%)	12 (11%)	0 (0%)
Smoked Pork	55 ( 65%)	20 (24%)	10 (12%)	59 ( 52%)	52 (46%)	3 (3%)
Bacon	83 ( 98%)	2 ( 2%)	0 ( 0%)	104 ( 91%)	10 ( 9%)	0 (0%)
Any Beef	84 ( 99%)	1 ( 1%)	0 ( 0%)	114 (100%)	0 ( 0%)	0 (0%)
Steak	84 ( 99%)	0 ( 0%)	1 ( 1%)	113 ( 99%)	1 ( 1%)	0 (0%)
Roast Beef	84 ( 99%)	0 ( 0%)	1 ( 1%)	110 ( 96%)	4 ( 4%)	0 (0%)
Hamburger	84 ( 99%)	1 ( 1%)	0 ( 0%)	111 ( 97%)	3 ( 3%)	0 (0%)
Veal	55 ( 65%)	24 (28%)	6 ( 7%)	76 ( 67%)	37 (32%)	1 (1%)

Hot Dogs	83 ( 98%)	1 ( 1%)	1 ( 1%)	103 ( 90%)	11 (10%)	0 (0%)
Bologna	65 ( 76%)	17 (20%)	3 ( 4%)	77 ( 68%)	37(32%)	0 (0%)
Salami	68 ( 80%)	15 (18%)	2 ( 2%)	80 ( 70%)	34 (30%)	0 (0%)
Sausage	80 ( 94%)	4 ( 5%)	1 ( 1%)	95 ( 83%)	19 (17%)	0 (0%)
Pepperoni	61 ( 72%)	20 (24%)	4 ( 5%)	65 ( 57%)	49 (43%)	0 (0%)
Kielbasa	52 ( 61%)	27 (32%)	6 ( 7%)	48 ( 42%)	65 (57%)	1 (1%)
Kishka	8 ( 9%)	67 (79%)	10 (12%)	8 ( 7%)	106 (93%)	0 (0%)
Scrapple	9 ( 11%)	57 (67%)	19 (22%)	14 ( 12%)	98 (86%)	2 (2%)
Sausessen	0 ( 0%)	68 (80%)	17 (20%)	0 ( 0%)	113 (99%)	1 (1%)
Meat Spreads	43 ( 51%)	37 (43%)	5 ( 6%)	56 ( 49%)	58 (51%)	0 (0%)
Poultry	85 (100%)	0 ( 0%)	0 ( 0%)	112 ( 98%)	2 ( 2%)	0 (0%)
Deer	53 ( 62%)	26 (31%)	6 ( 7%)	62 ( 54%)	52 (46%)	0 (0%)
Rabbit	42 ( 49%)	34 (40%)	9 (11%)	57 ( 50%)	57 (50%)	0 (0%)
“Other” Meat	35 ( 41%)	44 (52%)	6 ( 7%)	40 ( 35%)	74 (65%)	0 (0%)
“Other”	38 ( 45%)	38 (45%)	9 (11%)	35 ( 31%)	79 (69%)	0 (0%)
Canned Meat						
Any Brain	16 ( 19%)	64 (75%)	5 ( 6%)	29 ( 25%)	85 (75%)	0 (0%)
Any Tongue	28 ( 33%)	53 (62%)	4 ( 5%)	41 ( 36%)	72 (63%)	1 (1%)
Any Eyeball	0 ( 0%)	82 (96%)	3 ( 4%)	0 ( 0%)	114(100%)	0 (0%)
Any Heart	20 ( 24%)	58 (68%)	7 ( 8%)	29 ( 25%)	85 (75%)	0 (0%)
Any Kidney	10 ( 12%)	72 (85%)	3 ( 4%)	13 ( 11%)	100 (88%)	1 (1%)
Any Gizzard	6 ( 7%)	78 (92%)	1 ( 1%)	1 ( 1%)	113 (99%)	0 (0%)
Any Foot	23 (27%)	58 (68%)	4 ( 5%)	29 (25%)	85 (75%)	0 ( 0%)
Any Liver	70 (82%)	13 (15%)	2 ( 2%)	88 (77%)	26 (23%)	0 ( 0%)
Beef	59 (69%)	24 (28%)	2 ( 2%)	72 (63%)	42 (37%)	0 ( 0%)
Pork	6 ( 7%)	77 (91%)	2 ( 2%)	13 (11%)	101 (89%)	0 ( 0%)
Poultry	18 (21%)	65 (76%)	2 ( 2%)	8 ( 7%)	106 (93%)	0 ( 0%)
Any	18 (21%)	64 (75%)	3 (4%)	10 ( 9%)	102 (89%)	2 ( 2%)
Stomach/ Intestine						
Beef	13 (15%)	69 (81%)	3 (4%)	6 ( 5%)	106 (93%)	2 ( 2%)
Pork	6 ( 7%)	76 (89%)	3 ( 4%)	3 ( 3%)	109 (96%)	2 ( 2%)
Any Internal Organ	38 (45%)	44 (52%)	3 ( 4%)	30 (26%)	82 (72%)	2 ( 2%)
Tissue						
Beef	28 (33%)	54 (64%)	3 ( 4%)	18 (16%)	94 (82%)	2 ( 2%)
Pork	17 (20%)	65 (76%)	3 ( 4%)	16 (14%)	96 (84%)	2 ( 2%)
Poultry	9 (11%)	73 (86%)	3 ( 4%)	9 ( 8%)	102 (89%)	3 ( 3%)
Any Seafood	83 (98%)	2 ( 2%)	0 ( 0%)	112 (98%)	2 ( 2%)	0 ( 0%)
Canned	76 (90%)	7 ( 8%)	2 ( 2%)	105 (92%)	9 ( 8%)	0 ( 0%)
Fish						
Pickled Fish	24 (28%)	59 (70%)	2 ( 2%)	33 (29%)	81 (71%)	0 ( 0%)
Smoked	34 (40%)	49 (58%)	2 ( 2%)	39 (34%)	75 (66%)	0 ( 0%)
Fish						
Shrimp	69 (81%)	14 (17%)	2 ( 2%)	93 (82%)	21 (18%)	0 ( 0%)
Lobster	60 (71%)	23 (27%)	2 ( 2%)	68 (60%)	46 (40%)	0 ( 0%)
Crab	54 (64%)	25 (29%)	6 ( 7%)	68 (60%)	46 (40%)	0 ( 0%)
“Other”	33 (39%)	48 (56%)	4 ( 5%)	52 (46%)	62 (54%)	0 ( 0%)
Shell Fish						
Raw Fish	15 (18%)	67 (79%)	3 ( 4%)	12 (11%)	102 (89%)	0 ( 0%)
Raw Oyster	31 (36%)	50 (59%)	4 ( 5%)	35 (31%)	78 (68%)	1 ( 1%)
Raw Clam	3 ( 4%)	77 (91%)	5 ( 6%)	6 ( 5%)	108 (95%)	0 ( 0%)
Raw Shrimp	0 ( 0%)	79 (93%)	6 ( 7%)	4 ( 4%)	110 (96%)	0 ( 0%)
Rare/Raw Meat	21 (25%)	62 (73%)	2 ( 2%)	14 (12%)	93 (82%)	7 ( 6%)

* The 85 matched case-control pairs or triples are used: 56 cases had 1 matched control and 29 cases had 2 matched controls

**Table 3:** Odds ratios derived from exact matched analysis for food items likely to contain organ tissue and cooking preparation: ‘EVER’ vs ‘NEVER’

Food/Preparation	Odds Ratio 95% CI (2-Sided)	P-Value (1-Sided)
**Items Likely to Contain Organ Tissue**		
Hot Dogs	7.2 (1.0 – 315.6)	< 0.025
Bologna	1.7 (0.9 – 3.6)	< 0.07
Salami	1.9 (0.9 – 4.1)	< 0.055
Sausage	4.4 (1.2 – 24.3)	< 0.009
Pepperoni	2.4 (1.2 – 5.1)	< 0.008
Kielbasa	3.2 (1.6 – 7.0)	< 0.0003
Kishka	2.1 (0.6 – 8.2)	NS ( 0.17 )
Scrapple	1.04 (0.3 – 3.1)	NS ( 0.56 )
Sausessen	No Exposure	----
Meat Spreads	1.3 (0.7 – 2.4)	NS ( 0.25 )
“Other” Canned Meat	2.9 (1.4 – 6.5)	< 0.002

**Organ Tissue**		
Any Brain	0.7 (0.3 – 1.7)	NS (0.26)
Any Eyeball	No Exposure	----
Any Heart	1.0 (0.5 – 2.1)	NS (0.57)
Any Kidney	1.2 (0.4 – 3.4)	NS (0.47)
Gizzard (Poultry)	7.6 (0.9 – 354.8)	< 0.04
Any Liver	1.9 (0.8 – 4.9)	< 0.08
Beef Liver	1.7 (0.8 – 3.5)	< 0.09
Pork Liver	0.6 (0.2 – 1.8)	NS (0.21)
Poultry Liver	3.1 (1.2 – 9.0)	< 0.007
Any Stomach/Intestine	3.7 (1.3 – 13.1)	0.006
Beef Stomach/Intestine	4.5 (1.2 – 25.4)	0.01
Pork Stomach/Intestine	3.7 (0.6 – 37.6)	< 0.10
Any Internal Organ Tissue	2.5 (1.3 – 5.3)	< 0.004
Beef Internal Organ Tissue	3.2 (1.4 – 8.4)	< 0.003
Pork Internal Organ Tissue	1.6 (0.7 – 3.7)	NS (0.14)
Poultry Internal Organ Tissue	1.4 (0.5 – 3.9)	NS (0.34)

**Cooking Preparation**		
Rare/Raw Meat	2.0 (0.9 – 4.7)	< 0.05

NS: Not Significant

**Table 4:** Trend analyses of consumption for specific food items: ‘HIGH’, ‘LOW’ vs ‘NEVER’*

Food Item	Consumption	Odds Ratio; 95% CI (2-Sided)	P-VALUE (1-Sided)	TREND P-Value (2-Sided)
HOT DOGS	‘LOW’	6.1 (0.8 – 48.7)	< 0.05	< 0.009
	‘HIGH’	10.5 (1.2 – 88.7)	< 0.02	
SALAMI	‘LOW’	1.4 (0.6 – 2.8)	0.21	0.003
	‘HIGH’	3.5 (1.5 – 8.5)	< 0.003	
SAUSAGE	‘LOW’	3.9 (1.1 – 13.9)	< 0.02	< 0.02
	‘HIGH’	5.4 (1.4 – 20.0)	< 0.01	
PEPPERONI	‘LOW’	2.3 (1.1 – 4.6)	< 0.01	< 0.02
	‘HIGH’	2.6 (0.9 – 7.3)	< 0.04	
KIELBASA	‘LOW’	3.2 (1.6 – 6.2)	< 0.001	0.002
	‘HIGH’	6.5 (1.0+ – 41.0)	< 0.03	
BOLOGNA	‘LOW’	1.2 (0.6 – 2.4)	0.33	< 0.006
	‘HIGH’	2.9 (1.3 – 6.5)	< 0.005	

* Uses only cases with at least one control. Low: < 2 times/month; High: >= 2 times/month

**Table 5:** Comparison of reported rates of consumption of specific food items for controls and matched control-surrogates

Food Item	Concordance Rate	(Control Self-Response , Surrogate Response)*								
		(− , −)	(+ , +)	(− , +)	(+ , −)	(? , −)	(? , +)	(− , ?)	(+ , ?)	(? , ?)
Hot Dogs	0.78	1	24	3	2	0	0	0	2	0
Bologna	0.47	6	9	6	9	0	0	1	1	0
Salami	0.69	4	18	2	5	0	0	2	1	0
Sausage	0.78	1	24	3	3	0	0	0	1	0
Pepperoni	0.56	6	12	7	6	0	0	0	1	0
Kielbasa	0.59	10	9	3	7	0	1	1	1	0
Kishka	0.72	23	0	2	5	0	0	2	0	0
Scrapple	0.78	24	0	0	3	0	0	4	0	1
Meat Spreads	0.41	8	5	4	11	0	0	0	4	0
“Other” Canned Meat	0.56	13	5	7	6	0	0	0	1	0
Any Brain	0.59	17	2	3	9	0	0	1	0	0
Any Heart	0.66	19	2	3	6	0	0	1	1	0
Any Kidney	0.78	21	4	3	2	0	0	1	1	0
Any Liver	0.69	2	20	3	6	0	0	0	1	0
Any Stomach- Intestine	0.84	25	2	0	2	1	0	1	1	0

− = no consumption; + = consumption; ? = consumption unknown

**Table 6:** Comparison of matched and unmatched odds ratio estimates for food items likely to contain organ tissue and cooking preparation: ‘EVER’ vs ‘NEVER’

Food/Preparation	Control Self-Response Odds Ratios		Control-Surrogate Response Odds Ratios
	Exact Matched Analysis	Unmatched Analysis	Exact Matched Analysis
Hot Dogs	7.2**	5.7**	∞
Bologna	1.7*	2.3**	5.4**
Salami	1.9*	2.1**	1.6
Sausage	4.4*	4.1**	3.6
Pepperoni	2.4**	2.2**	3.8*
Kielbasa	3.2**	2.4**	3.9**
Kishka	2.1	1.4	3.0
Scrapple	1.04	0.9	∞
Sausessen	No Exposure		
Meat Spreads	1.3	1.1	2.8*
“Other” Canned Meat	2.9**	2.3**	3.3**
Any Brain	0.7	0.9	2.5
Any Eyeball	No Exposure		
Any Heart	1.0	1.3	1.0
Any Kidney	1.2	1.5	0.2
Gizzard	7.6**	9.4**	No Exposure
Any Liver	1.9*	2.0**	1.8
Beef Liver	1.7*	1.6**	1.1
Pork Liver	0.6	1.0	No Exposure
Poultry Liver	3.1**	4.0**	8.0**
Any Stomach/Intestine	3.7**	3.3**	∞ **
Beef Stomach/Intestine	4.5**	2.9**	∞
Pork Stomach/Intestine	3.7*	4.2**	3.0
Any Internal Organ	2.5**	2.6**	∞ **
Beef Organ	3.2**	2.9**	6.0**
Pork Organ	1.6	1.8**	∞ **
Poultry Organ	1.4	1.9*	2.0
Rare/Raw Meat	2.0**	1.9*	2.2

* 0.05 < p ≤ 0.1

** p ≤ 0.05

**Table 7:** Absolute and relative frequencies of consumption of beef, brain and venison for cases, ‘non-cases’ and controls by time period: National CJD Surveillance Unit Reports 1995, 1997 [54,55]

Items	Frequency of Consumption	N of Cases (%)		N of ‘Non-Cases (%)		N of Controls (%)	

		1990-1995	1995-1997	1990-1995	1995-1997	1990-1995	1995-1997
Beef	< Monthly	10 ( 6.8%)	7 (13.2%)	2 (4.2%)	4 (14.8%)	25 (16.9%)	9 (16.4%)
	≥ Monthly*	62 (41.9%)	22 (41.5%)	19 (39.6%)	11 (40.7%)	63 (42.6%)	25 (45.5%)
	≥ Weekly	76 (51.4%)	24 (45.3%)	27 (56.3%)	12 (44.4%)	60 (40.5%)	21 (38.2%)
	Totals	148	53	48	27	148	55
	P-Values	P = 0.16**		P = 0.18**		P = 1.0**	
Brain***	Never	123 (86.9%)	44 (86.3%)	68 (90.7%)		141 (94.6%)	52 (92.9%)
	< Yearly	15 (10.5%)	5 ( 9.8%)	5 ( 6.7%)		6 ( 4.0%)	4 ( 7.1%)
	≥ Yearly	5 ( 3.5%)	2 ( 3.9%)	2 ( 2.7%)		2 ( 1.3%)	0 ( 0.0%)
	Totals	145	51	75		149	56
	P-Values	P = 1.0**		–		P = 0.74**	
Venison***	Never	101 (69.2%)	35 (64.8%)	40 (80.0%)		125 (83.3%)	35 (62.5%)
	< Yearly	33 (22.6%)	14 (25.9%)	9 (18.0%)		23 (15.3%)	19 (33.9%)
	≥ Yearly	12 ( 8.2%)	5 ( 9.3%)	1 ( 2.0%)		2 ( 1.3%)	2 (3.6%)
	Totals	146	54	50		150	56
	P-Values	P = 0.61**		–		P = 0.002**	

* But not as frequently as weekly

** < Monthly vs ≥ Monthly for Beef; Never vs Ever for Brain and Venison

*** Includes ‘non-cases’ for 1990-1997

**Table 8:** Comparison of the findings from the published studies with the present study

Food item: “ever exposed”	Bobowick et al. [7]	Kondo & kuroiwa [9]	Davanipour et al. [5]	Harries- Jones et al. [10]	Van duijn et al. [11]	Ruegger et al. [12] ^†^	Present Study
Brain	OR < 1.4	No Exp.	OR = 0.7	OR ≈ 1.0	OR = 1.7**	OR = 0.51	OR = 0.7
Kidney	xxx	xxx	OR = 0.7	OR < 1.0	OR = 1.1	OR = 1.96**	OR = 1.2
Liver	xxx	xxx	OR = 5.9*	OR ≈ 1.0	OR = 1.2	OR = 1.23	OR = 1.9
Stomach/Intestine	xxx	xxx	OR = 1.0	OR > 1.0	OR = 1.0	OR = 1.32^	OR = 3.7**
Beef	xxx	xxx	All Exposed	xxx	OR ≈ 1.7	OR = 0.88	yyy
Veal	xxx	xxx	OR = 1.0	xxx	OR ≈ 1.2	OR = 1.55	OR = 1.2
Lamb	xxx	xxx	OR = 1.3	xxx	OR ≈ 1.0	OR = 0.74	OR = 0.8
Pork	xxx	xxx	OR = 4.5	xxx	OR ≈ .3***	OR = 0.93	OR = 5.4***
Raw/rare meat^#^	OR ≈ 1.0	OR ≈ 1.0	OR = 12.5**^,††^	xxx	OR = 16*	OR = 0.84	OR = 2.0**

xxx No data available

yyy All controls and all but 1 case exposed

* Marginally statistically significant (p < 0.10)

** Statistically significant (p < 0.05)

*** Statistically significant trend in risk with increased consumption

# Raw/rare meat consumption was used in the earlier Davanipour et al. [5] and the present studies; labeled “tartare/carpaccio”, which may also contain raw fish, in Ruegger et al. [12]

^ Labeled “innards” in Ruegger et al. [l^2^]

† Ruegger et al. [12] found consumption of “hamburgers at restaurants ever” to have an adjusted OR=1.74, p=0.086

†† The OR is for hospital controls, with a p< 0.01; for hospital and family controls combined, OR=23.2, p<0.01

## ABBREVIATIONS

AD: Alzheimer’s disease
BASE: bovine amyloidotic spongiform encephalopathy
BSE: bovine spongiform encephalopathy
CJD: Creutzfeldt-Jakob disease
MDNV: motor dorsal nucleus of the vagus
ME7: a mouse-adapted scrapie strain
M/V: methionine/valine polymorphism
M/V2 CJD: a particular strain of sCJD
vCJD: variant CJD, transmitted from cattle to humans
OR: odds ratio
PrP^Sc^: infectious agent for prion diseases; Sc stands for scrapie, the name of the disease in sheep
RML: a mouse-adapted scrapie strain
sCJD: sporadic Creutzfeldt-Jakob disease
Tga20: a specific transgenic mouse model
TME: transmissible mink encephalopathy
TSE: transmissible spongiform encephalopathy
VaD: vascular dementia
